# The role of Na^+^/H^+ ^exchanger in Ca^2+ ^overload and ischemic myocardial damage in hearts from type 2 diabetic *db/db *mice

**DOI:** 10.1186/1475-2840-11-33

**Published:** 2012-04-11

**Authors:** Ryuko Anzawa, Shingo Seki, Tomohisa Nagoshi, Ikuo Taniguchi, Danielle Feuvray, Michihiro Yoshimura

**Affiliations:** 1 Division of Cardiology, Department of Internal Medicine, The Jikei University School of Medicine, 3-25-8 Nishi-Shimbashi, Minato-ku, Tokyo 105-8461, Japan; 2 University of Paris-Sud 11 and UMR-CNRS 8078, Le Plessis Robinson, France

## Abstract

**Background:**

A higher increase in intracellular Na^+ ^via Na^+^/H^+ ^exchanger (NHE) during ischemia has been reported in type 2 diabetic mouse hearts. We investigated the role of NHE in inducing changes in cytoplasmic Ca^2+ ^concentration ([Ca^2+^]_i_) and alterations in ventricular function during ischemia-reperfusion in type 2 diabetic mouse hearts.

**Methods:**

Hearts from male type 2 diabetic *db/db *(12-15 weeks old) and age-matched control *db/+ *mice were subjected to Langendorff perfusion and loaded with 4μM of the Ca^2+ ^indicator fura-2. The hearts were exposed to no-flow ischemia for 15 minutes and then reperfused. [Ca^2+^]_i _was measured by monitoring fura-2 fluorescence at 500 nm (excitation wavelengths of 340 and 380 nm), while left ventricular (LV) pressure was simultaneously measured.

**Results:**

*db/db *hearts exhibited a lower recovery of LV developed pressure than *db/+ *hearts during reperfusion following ischemia. Diastolic [Ca^2+^]_i _was increased to a greater level in diabetic hearts than in the control hearts during ischemia and reperfusion. Such an increase in cytoplasmic Ca^2+ ^overload during ischemia-reperfusion in diabetic hearts was markedly reduced in the presence of the NHE inhibitor cariporide. This was accompanied by a significantly improved recovery of ventricular function on reperfusion, as shown by a lower increase in diastolic pressure and increased recovery of developed pressure.

**Conclusion:**

NHE plays a key role in enhancing cytoplasmic Ca^2+ ^overload during ischemia-reperfusion and severely impairing post-ischemic cardiac function in hearts from type 2 diabetic *db/db *mice.

## Background

Diabetes mellitus (DM) has been reported to be an independent predictor of cardiovascular morbidity and mortality in patients with ischemic heart disease [1], as well as heart failure [2], while direct deleterious effects of DM on hearts also have been reported [3]. However, few investigations of the cardiac consequences of type 2 diabetes, particularly in regard to sensitivity to ischemia, have been performed [4-6]. It is recognized that cytoplasmic Ca^2+ ^overload is an important mechanism in myocardial ischemic injury [7-10]. Cytoplasmic Ca^2+ ^overload occurs via a combination of activities of Na^+^/H^+ ^exchanger (NHE) and Na^+^/Ca^2+ ^exchanger (NCX), with the latter functioning in the reverse mode [9-13]. Changes in cytoplasmic Ca^2+ ^concentration ([Ca^2+^]_i_) during ischemia have not been evaluated in type 2 diabetic hearts. We previously reported that a highly increased intracellular Na^+ ^concentration ([Na^+^]_i_), mainly via NHE, caused enhanced sensitivity to ischemia in type 2 diabetic *db/db *mouse hearts [5]. Therefore, we speculated that NHE plays a key role in cytoplasmic Ca^2+ ^overload in ischemic cardiomyocytes of type 2 diabetic hearts.

The purpose of the present study was to determine changes in diastolic [Ca^2+^]_i _and Ca^2+ ^transient amplitude during ischemia and reperfusion in isolated type 2 diabetic *db/db *mouse hearts loaded with fura-2, a fluorescent dye [8,14]. The correlation between [Ca^2+^]_i _and cardiac function was investigated, as well as the importance and role of NHE in [Ca^2+^]_i _changes.

## Methods

### Experimental animals

This study was conducted in accordance with the guidelines of the Guidance Committee of the Jikei University School of Medicine for the Use and Care of Animals, and conformed to the Guidelines for the Care and Use of Laboratory Animals published by the US National Institutes of Health (NIH Publication No. 85-23, revised 1996). Male BKS. Cg- + *Lepr^db^/+Lepr^db^*/Jcl (*db/db*) mice and their non-diabetic heterozygous control littermates BKS. Cg-m+/+*Lepr^db^*/Jcl (*db/+*) mice were purchased from CLEA Japan (Tokyo, Japan). All animals used in this study were males between 12 and 15 weeks of age. The animals were housed in groups (4 or 5) and given free access to food and water.

### Isolated perfused heart preparation

Mice were anesthetized with sodium pentobarbital (100 mg/kg) and heparinized (100U) i.p. Each heart was then quickly removed and the ascending aorta was cannulated with a 19-gauge steel cannula. Langendorff's retrograde perfusion (80 mmHg perfusion pressure) was started with N-2-hydroxy-ethylpiperazine-N'-2-ethansulfonic acid (HEPES)-buffered Tyrode's solution (140 mM NaCl, 6 mM KCl, 1 mM MgCl_2_, 2 mM CaCl_2_, 10 mM HEPES, 10 mM glucose, gassed with 100% O_2_, pH 7.4, 37°C). Hearts were not perfused in the presence of fatty acids [15,16]. All perfusion solutions were filtered prior to use. Isovolumic left ventricular developed pressure (LVDP), heart rate, and maximum and minimum first derivatives of developed pressure (dP/dt max and dP/dt min) were monitored using a fluid-filled polyvinylchloride film balloon inserted into the left ventricle via the mitral valve. The balloon was connected to a Statham 23 ID pressure transducer and polygraph system (Nihon Koden Co., Japan). The initial left ventricular end diastolic pressure (LVEDP) was adjusted to 0 mmHg. LVDP was calculated by subtracting LVEDP from systolic pressure. Pressure signals were obtained using a PowerLab data acquisition system (AD Instruments Japan, Tokyo, Japan). The hearts were allowed to contract spontaneously in all the experiments.

### Fluorescence measurements

Intracellular Ca^2+ ^levels were measured by the use of the cell-permeable Ca^2+ ^sensitive fluorescent dye, fura-2/acetoxymethyl ester (AM) (Dojindo Laboratories, Japan), with an optical-fiber analysis system (CAF-110, CA-200DO; Japan Spectroscopic Co., Japan) [8,9]. Following 30 minutes of stabilization, the heart was loaded for 30 minutes by simple perfusion in constant flow system that maintained a coronary flow rate of ≃ 2.2-2.5 ml/minute with a peristaltic pump. The loading buffer (Tyrode's solution) contained 4 μM of fura-2/AM and was given for 30 minutes. During the subsequent 20 minutes, the heart was perfused with normal Tyrode's solution to wash out the dye in the extracellular space. The fluorescent dye was dissolved in dimethyl sulfoxide containing Cremophor EL (25% w/v). Excitation light from a Xe lamp was transferred via the fiber onto the epicardial surface of the LV and the fluorescent light was collected through another fiber onto a photomultiplier. The ratio of 500 nm fluorescence intensity (F_340 _/F_380_) excited at 340 and 380 nm of UV light was monitored as a quantitative index of [Ca^2+^]_i_, and was independent of the fura-2 concentration. Background autofluorescence was subtracted to assess diastolic [Ca^2+^]_i_. We evaluated changes in diastolic [Ca^2+^]_i _by using the following equation to equalize the differences in fura-2 loading conditions in each heart.

$$\text{Changes in diastolic }{\left[\text{C}{\text{a}}^{\text{2}+}\right]}_{\text{i}}\left(\%\right)=\left[\text{R}-{\text{R}}_{\text{pre}}\right]/{\text{A}}_{\text{pre}}\times \text{1}00$$

Where, R is the diastolic ratio, R_pre _is the pre-ischemic diastolic ratio and A_pre _is the amplitude of pre-ischemic ratio. Each ratio was calculated from the value of fluorescence intensity at 340 nm (autofluorescence subtracted) divided by fluorescence intensity at 380 nm (autofluorescence subtracted). Each ratio was expressed in arbitrary units. Calibration of fura-2 was not performed. Throughout the experiment, we simultaneously monitored fura-2 fluorescence and hemodynamic parameters.

### Experimental protocol

Based on results from pilot experiments, the experimental protocol consisted of 10 minutes of control perfusion, followed by 15 minutes of no-flow ischemia and 15 minutes of reperfusion. In each group, diabetic and non-diabetic hearts either received or did not receive the NHE inhibitor cariporide (1 μmol/l; a gift from Aventis Pharma) [5,11,13,17,18]. When present, cariporide was added to the perfusion solution at 10 minutes before inducing ischemia and remained throughout.

### Analysis of plasma metabolites

Blood samples (fed dietary status) were taken from the body cavity after excision of the heart. Plasma glucose was measured using a glucose oxidase method.

### Statistical analysis

Results are presented as the means ± SEM. The data were analyzed using either Student's t test for unpaired data or ANOVA, followed by the appropriate post hoc test, using Scheffe's test to locate differences between groups. Significance was set at *p *< 0.05.

## Results

### Animal characteristics

At 12-15 weeks of age, body weights and plasma glucose levels in diabetic *db/db *mice were significantly higher than those in non-diabetic *db/+ *littermates (Table 1). These observations are in agreement with those of previous studies [4,5,17,19].

**Table 1:** Characteristics of control (*db/+*) and diabetic (*db/db*) mice


	***db/+***	***db/db***
	**(n = 17)**	**(n = 17)**

**Body weight (g)**	29.4 ± 0.5	51.0 ± 1.0 *

**Plasma glucose (mg/dl)**	226.1 ± 8.0	615.6 ± 10.1**

Values are the means ± SE. * *p *< 0.05, ** *p *< 0.01 vs. *db/+ *mice

### Pilot experiments

In pilot experiments without the ischemia-reperfusion protocol, a decrease of LVDP by 12% was observed after fura-2 loading and the washing out process. Bleaching of fura-2 was observed after 60 minutes of perfusion. Fluorescence intensities and ratios were stable, and did not decrease during at least 50 minutes of perfusion.

When the initial LVEDP was adjusted to 10 mmHg, LVDP tended to decrease after 50 minutes of perfusion in both *db/db *and *db/+ *hearts. However, when that was adjusted to 0 mmHg, a stable LVDP time course was observed during the 50 minutes of perfusion.

Several durations of ischemia were assessed after fura-2 loading and the washing out process. Following only 10 minutes of ischemia cardiac function recovered well in both diabetic and control groups, whereas poor function recovery was observed after ischemic periods longer than 20 minutes. All of the following experiments were therefore performed with ischemia duration of 15 minutes.

### Ventricular function and [Ca^2+^]_i _during control perfusion in hearts from diabetic *db/db *mice

**Figure 1:**
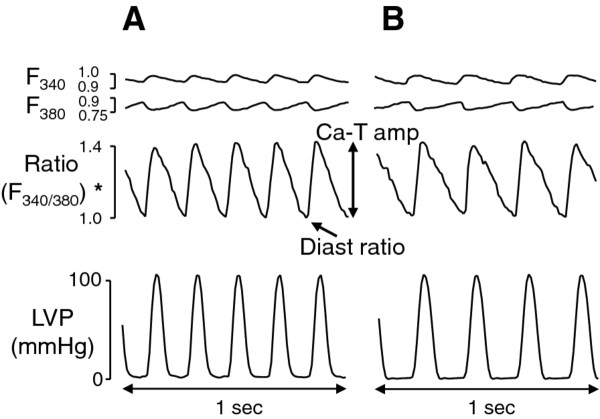
**Original traces showing F340 and F380, F340/F380 ratio and left ventricular pressure in hearts from control *db/+ *(A) and diabetic *db/db *(B) mice**. Ca-T amp, Ca^2+ ^transient amplitude; Diast ratio, diastolic fura-2 ratio; LVP, left ventricular pressure. *Autofluorescence not subtracted.

Original trace recordings of fura-2 fluorescence, ratios and LVP during control perfusion are illustrated in Figure 1. Autofluorescence was not subtracted. Ca^2+ ^recordings revealed phasic changes with steep upstrokes and slow declines.

Systolic and diastolic levels of the F340/F380 ratio were stable, indicating that heart movements did not affect these levels. LVDP measured after 30 minutes of perfusion was in the range of values previously reported [5] for mouse hearts under similar conditions, with no difference between control and diabetic hearts that received or not cariporide (Table 2). Likewise, cariporide did not influence heart rate or calcium measurements in both *db/db *and *db/+ *mouse hearts. Also, diastolic fura-2 fluorescence ratio and Ca^2+ ^transient amplitude showed similar levels in both groups (Table 2). However, the duration of Ca^2+ ^transients was increased in diabetic *db/db *hearts compared with that in non-diabetic *db/+ *hearts, as revealed by time-to-peak transient values and duration at 50% recovery. Differences in Ca^2+ ^transients between diabetic and non-diabetic hearts remain unchanged in the presence of the NHE inhibitor cariporide. Such an observation is consistent with previous observations of altered calcium handling in ventricular myocytes from diabetic animals resulting primarily from dysfunction of the sarcoplasmic reticulum [20].

**Table 2:** Hemodynamic parameters and calcium measurements during control perfusion in non-diabetic and diabetic hearts


	***db/+***	***db/db***	***db/+*** **cariporide**	***db/db*** **cariporide**
	**(n = 9)**	**(n = 9)**	**(n = 8)**	**(n = 8)**

LVDP (mmHg)	103.9 ± 4.9	103.5 ± 8.5	104.2 ± 5.6	102.4 ± 7.8
Heart rate (bpm)	370.3 ± 16.2	309.6 ± 25.0*	358.6 ± 16.8	307.9 ± 18.9
dP/dt max (mmHg/ms)	3.70 ± 0.20	2.98 ± 0.33	3.71 ± 0.30	2.96 ± 0.20
dP/dt min (mmHg/ms)	3.04 ± 0.14	2.35 ± 0.21*	3.06 ± 0.25	2.34 ± 0.18
Diast ratio (a.u.)	1.31 ± 0.04	1.37 ± 0.02	1.32 ± 0.08	1.38 ± 0.05
Ca-T amp (a.u.)	0.60 ± 0.04	0.51 ± 0.03	0.59 ± 0.06	0.50 ± 0.07
TTP (ms)	37.5 ± 1.4	45.8 ± 5.1*	38.8 ± 1.3	45.6 ± 3.3
DT50 (ms)	62.1 ± 2.0	81.1 ± 4.7*	63.0 ± 1.4	82.3 ± 4.2
DT100 (ms)	128.1 ± 4.3	150.1 ± 14.6	130.4 ± 4.1	149.8 ± 11.5

Values are the means ± SE. LVDP, left ventricular developed pressure; dP/dt max and dP/dt min, maximum and minimum first derivatives of developed pressure; Diast ratio, diastolic fura-2 fluorescence ratio; Ca-T amp, amplitude of Ca^2+ ^transient; a.u., arbitrary unit; TTP, time-to-peak Ca^2+ ^transient; DT50, 50% decay-time of Ca^2+ ^transient, DT100, 100% decay-time of Ca^2+ ^transient. Background autofluorescence was subtracted for fura-2 fluorescence study. * *p *< 0.05 vs *db/+ *mice

### Ventricular function during ischemia and after reperfusion in hearts from diabetic *db/db *mice

**Figure 2:**
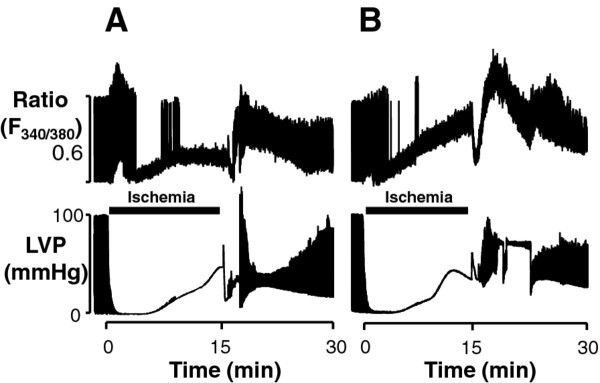
**Original traces showing F340/F380 ratio and left ventricular pressure during ischemia and reperfusion in hearts from *db/+ *(A) and *db/db *(B) mice**.

**Figure 3:**
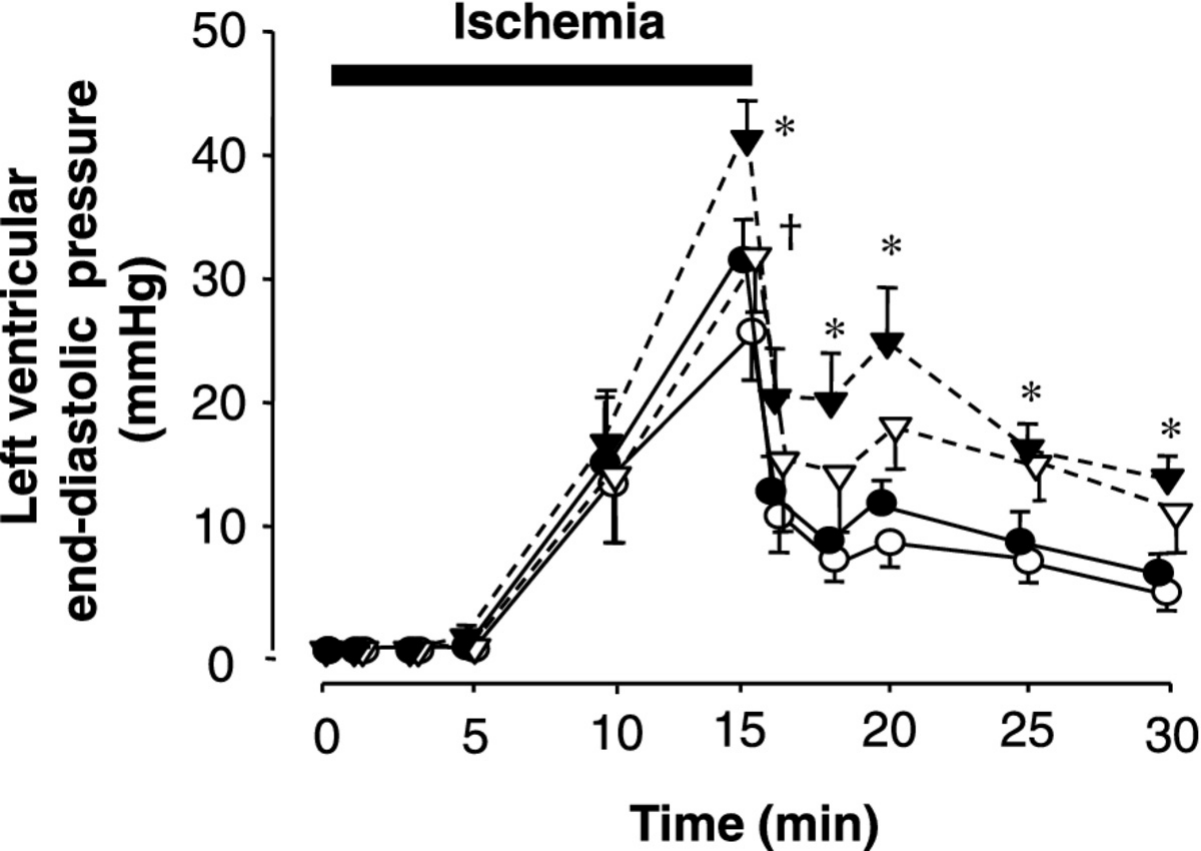
**Changes in left ventricular end-diastolic pressure during ischemia and reperfusion in hearts from non-diabetic *db/+ *mice (closed circles, n = 9), diabetic *db/db *mice (closed triangles, n = 9), non-diabetic *db/+ *mice that received cariporide (open circles, n = 8), and diabetic *db/db *mice that received cariporide (open triangles, n = 8)**. *p < 0.05 vs. *db/+*, ^†^*p *< 0.05 vs. without cariporide.

**Figure 4:**
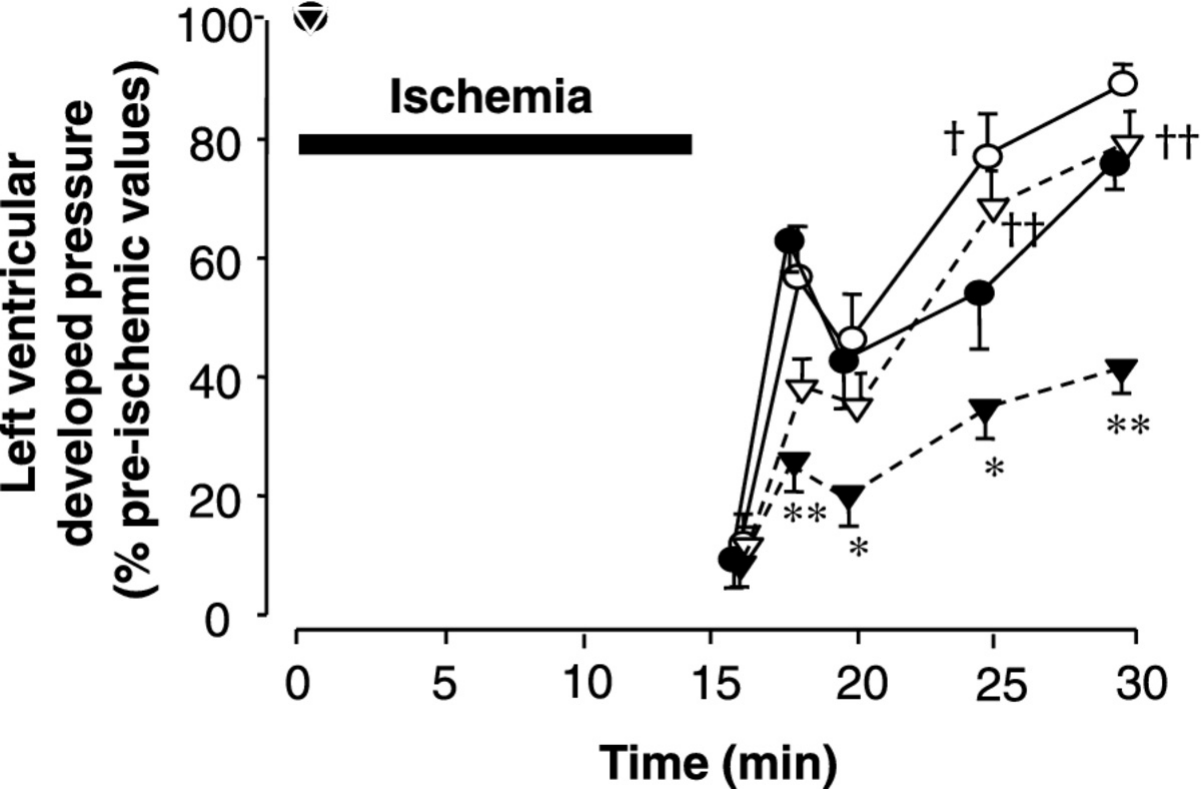
**Percent changes in left ventricular developed pressure during ischemia and reperfusion in hearts from non-diabetic *db/+ *mice (closed circles, n = 9), diabetic *db/db *mice (closed triangles, n = 9), non-diabetic *db/+ *mice that received cariporide (open circles, n = 8), and diabetic *db/db *mice that received cariporide (open triangles, n = 8)**. *p < 0.05 vs. *db/+*, **p < 0.01 vs. *db/+*, ^†^*p *< 0.05 vs. without cariporide, ^††^*p *< 0.01 vs. without cariporide.

After inducing ischemia, LVDP decreased to 0 over 1-2 minutes in hearts from both diabetic *db/db *and *db/+ *mice, and LVEDP increased (Figures 2, 3, 4). At the end of ischemia, LVEDP in diabetic *db/db *hearts was significantly higher (41.3 ± 3.1 mmHg) compared with that in control *db/+ *hearts (31.4 ± 2.8 mmHg, *p *< 0.05, Figure 3). Cariporide did not significantly affect the rise in LVEDP in *db/+ *hearts during ischemia and reperfusion (Figure 3), whereas it significantly reduced it in *db/db *hearts at the end of ischemia (Figure 3). Figure 4 shows the recovery rate of LVDP, which was significantly lower in *db/db *hearts compared with that in *db/+ *hearts throughout reperfusion (42.5 ± 3.8% and 81.2 ± 3.0%, respectively, at 15 minutes of reperfusion, *p *< 0.01). Cariporide improved the recovery of LVDP in both groups of hearts (Figure 4). However, the positive effect of cariporide was clearly significant only in hearts from diabetic *db/db *mice (80.5 ± 5.2% in the presence of cariporide vs. 42.5 ± 3.8% in its absence, Figure 4). In the present study we did not record electrocardiogram, which we had done previously [5], thus the influence of arrhythmia is unclear.

### Changes in [Ca^2+^]_i _during ischemia and after reperfusion in hearts from diabetic *db/db* mice

Figure 2 illustrates typical original traces obtained from individual hearts throughout the experiment. Background autofluorescence was not subtracted in any trace showing fura-2 ratio. In hearts from both non-diabetic *db/+ *(Figure 2A) and diabetic *db/db *(Figure 2B) mice, diastolic ratio levels elevated gradually during ischemia and additional elevations were observed immediately upon reperfusion. These ratios tended to return to pre-ischemic values during reperfusion. The amplitude of Ca^2+ ^transients rapidly decreased during ischemia in both heart groups. A progressive increase was observed during reperfusion.

**Figure 5:**
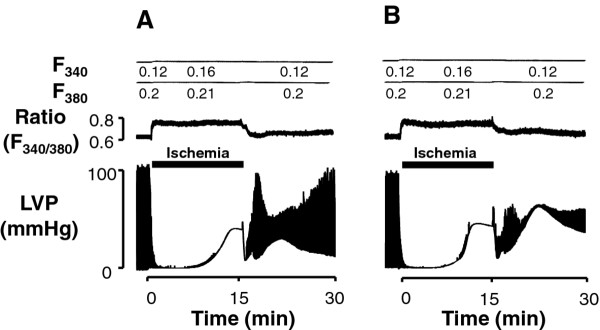
**Original traces showing F340 and F380, F340/F380 ratio and left ventricular pressure in hearts from fura-2 unloaded *db/+ *(A) and *db/db *(B) mice**.

We also investigated the effects of ischemia and reperfusion on myocardial autofluorescence. It has been demonstrated that the majority of autofluorescence is due to the intracellular metabolite NAPDH [21]. Figure 5 shows original traces of fluorescence changes at 340 and 380 nm, as well as their ratio, from unloaded hearts of both *db/+ *and *db/db *mice. The ratio of fluorescence was also markedly increased during ischemia and then decreased to the baseline value after reperfusion. It should be noted that fluorescence excited at 340 or 380 nm did not change significantly throughout the experiment. In the unloaded hearts, the ischemia-induced alteration of each fluorescence intensity (n = 4) was smaller compared with the loaded hearts. However, the increase in autofluorescence at 340 nm was larger than that at 380 nm after induction of ischemia, though the value for each remained constant during ischemia. The average values (arbitrary units) from 4 hearts in each groups were as follow: (i) 0.12 and 0.20, at 340 and 380 nm, respectively, during control perfusion and reperfusion, (ii) 0.16 and 0.21, at 340 and 380 nm, respectively, during ischemia. Therefore, the change in diastolic level of fluorescence ratio might have been overestimated during the early phase of ischemia when autofluorescence was not subtracted. It has been previously found that autofluorescence at both 340 and 380 nm increased in parallel during ischemia, with the result that the ratio remained constant [22]. In the present study, all hearts showed a large amplitude of fluorescence ratios, which likely limited any effect of changes in autofluorescence.

**Figure 6:**
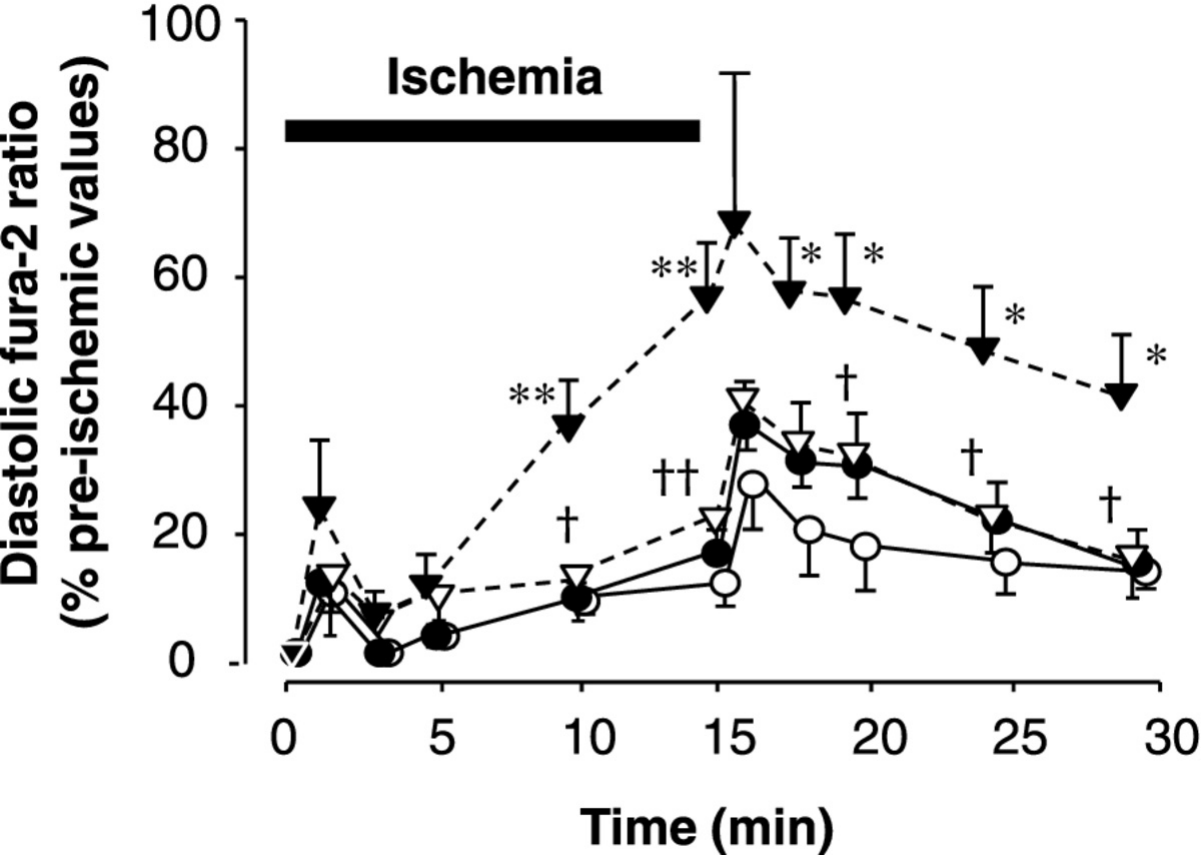
**Changes in diastolic fura-2 ratio during ischemia and reperfusion in hearts from non-diabetic *db/+ *mice (closed circles, n = 9), diabetic *db/db *mice (closed triangles, n = 9), non-diabetic *db/+ *mice that received cariporide (open circles, n = 8), and diabetic *db/db *mice that received cariporide (open triangles, n = 8)**. *p < 0.05 vs. *db/+*, **p < 0.01 vs. *db/+*, **^†^***p *< 0.05 vs. without cariporide, **^††^***p *< 0.01 vs. without cariporide.

Figure 6 shows changes in diastolic fura-2 ratio during ischemia and reperfusion. Diastolic fura-2 ratio increased more rapidly during no-flow ischemia in hearts from diabetic *db/db *mice than in those from control non-diabetic *db/+ *mice, reaching 56.0 ± 7.2% and 15.0 ± 2.6%, respectively, of the control values (*p *< 0.01) at the end of ischemia. Throughout reperfusion, diastolic fura-2 ratios remained elevated in hearts from diabetic *db/db *mice and were significantly higher than those in hearts from non-diabetic *db/+ *mice. Cariporide treatment markedly reduced the elevation in diastolic fura-2 ratio in hearts from diabetic *db/db *mice, during both ischemia and reperfusion.

## Discussion

The main finding obtained in the present study was a marked decrease in cytoplasmic Ca^2+ ^overload during ischemia-reperfusion in hearts from diabetic *db/db *mice in the presence of the NHE inhibitor cariporide. This was accompanied by significantly improved recovery of ventricular function on reperfusion, as assessed from our findings of a lower increase in diastolic pressure and increased recovery of developed pressure.

In agreement with previous reports that used mice under similar experimental conditions (i.e., same age and similar control perfusion conditions) [4-6], we found no difference in ventricular function between control and diabetic hearts (Table 2). Basal alterations in [Ca^2+^]_i _in hearts from diabetic *db/db *mice have been reported by several investigators. Belke et al. noted increased Ca^2+ ^leakage from the sarcoplasmic reticulum (SR) [19], while impaired [Ca^2+^]_i _cycling due to reductions in expressions of both sarcolemmal Ca^2+ ^channels and SR Ca^2+ ^release channels were demonstrated by Pereira et al. [23]. In the present study, during the control perfusion period, time to peak and decay time of Ca^2+ ^transient were significantly prolonged in *db/db *hearts (Figure 1 and Table 2). Our observations are in agreement with the above reports and represent a consistent feature of ventricular myocytes in diabetic hearts [20]. It has been shown that increased saturated fatty acid levels impair Ca^2+ ^handling and contraction in a reactive oxygen species (ROS)-dependent manner in normal cardiomyocytes [15]. However, it remains to be determined whether increased fatty acid utilization interferes with Ca^2+ ^handling in diabetic *db/db *mouse hearts. Basal alterations in [Ca^2+^]_i _might precipitate cytoplasmic Ca^2+ ^overload during ischemia-reperfusion, as we observed in the present *db/db *hearts. On the other hand, cytoplasmic Ca^2+ ^overload in these hearts was largely prevented by the presence of the NHE inhibitor cariporide (Figure 6). This finding indicates a major role for the ionic exchanger NHE in Ca^2+ ^overload. It is known that increases in [Na^+^]_i _in ischemic cardiomyocytes [5] generate Ca^2+ ^loading via reverse Na^+^/Ca^2+ ^exchanger, which in turn mediates much of the damage incurred upon reperfusion [24]. In this context azelnidipine [25] and ranolazine [26] have also been shown to be efficient at reducing intracellular calcium accumulation.

NHE is a key element in the physiological response of [Ca^2+^]_i _and Ca^2+ ^signaling in cardiomyocytes [27-29], and its activation has been reported to be correlated with cardiac hypertrophy via this system [17,27,29]. The present results are in line with our previous report that showed higher [Na^+^]_i _increase during ischemia and severely impaired post-ischemic cardiac function in hearts from diabetic *db/db *mice. In that study, NHE inhibitor cariporide reduced ischemia-induced [Na^+^]_i _increase and improved post-ischemic cardiac function in *db/db *hearts [5]. Likewise, elevation of diastolic fura-2 ratio in the present study was attenuated during ischemia and reperfusion and functional alterations on reperfusion were markedly reduced by cariporide in diabetic *db/db *hearts. Taken together, our previous [5] and present results suggest that the role of NHE is more important for ischemia-induced cytoplasmic Ca^2+ ^overload and myocardial damage in diabetic *db/db *hearts than in control *db/+ *hearts. Moreover, enhanced NHE activity during ischemia in *db/db *hearts can be inferred from these results. Indeed, enhanced NHE activity in ventricular myocytes has been reported in another genetic model of type 2 diabetes, the Goto-Kakizaki rat [17]. In addition, the use of HCO_3_^- ^free, HEPES buffered perfusion solution might have enhanced the contribution of NHE1 in the present study [30]. Among known factors stimulating cardiac NHE1 activity are [Ca^2+^]_i _[31,32] and angiotensin II [33-35]. In ventricular myocytes of diabetic *db/db *mice, NHE1 activity might be stimulated by basal alterations in [Ca^2+^]_i_, due to increased Ca^2+ ^leakage [18]. Future work will have to examine NHE1 activity in cardiac myocytes of diabetic *db/db *mice.

Inhibition of an NHE isoform located in the mitochondrial membrane and reduction of mitochondrial Ca^2+ ^([Ca^2+^]_m_) overload by one of the NHE blockers during ischemia-reperfusion have been reported [36]. We cannot exclude the possibility that cariporide may have exerted some effects on mitochondrial NHE and [Ca^2+^]_m _in the present study. In addition, though these effects of cariporide might be different between diabetic and non-diabetic hearts, [Ca^2+^]_m _cannot be selectively measured with the methods employed in this study. Finally, NHE inhibitors have recently been shown to affect mitochondria by blunting MPTP formation and ROS release [37]. An overproduction of mitochondrial ROS has indeed been shown in the heart in obesity related diabetes [38]. Further studies are still needed to investigate the role of ROS formation in ischemia-induced calcium overload in hearts of diabetic *db/db *mice.

Concerning clinical applications, blockade of NHE may provide salutary effects for diabetic patients with ischemic heart disease through an interaction with PKB/Akt [39,40] and other mechanisms [29,31-35]. However, it is unfortunate that the majority of trials conducted to test the effects of NHE inhibitors in ischemic heart disease cases have failed [41]. Intrinsically, valuable actions of NHE might be inhibited excessively in those trials. Hence, the methods or timings to inhibit NHE activity should be studied. For example, partial inhibition of aldosterone-induced genomic synthesis of NHE by eplerenone [42] may be an important choice.

## Conclusion

Cytosolic Ca^2+ ^overload during ischemia was much greater in hearts from type 2 diabetic *db/db *mice than in those from non-diabetic mice, which was accompanied by more severe post-ischemic ventricular dysfunction. Such marked effects of ischemia in diabetic hearts vanished in the presence of NHE inhibition. This study highlights an important role of NHE for Ca^2+ ^handling disturbances and resulting cardiac damage, particularly in diabetic hearts.

## Abbreviations

DM: Diabetes mellitus; NHE: Na^+^/H^+ ^exchanger; NCX: Na^+^/Ca^2+ ^exchanger; [Ca^2+^]_i_: Cytoplasmic Ca^2+ ^concentration; [Na^+^]_i_: Intracellular Na^+ ^concentration; LVDP: Left ventricular developed pressure; LVEDP: Left ventricular end diastolic pressure

## Competing interests

The authors declare that they have no competing interests.

## Authors' contributions

RA conceived of the study, carried out the experiments, and drafted the manuscript. SS and TN participated in the fluorescence measurement study. IT, DF, and MY conceived of the study, participated in its design and coordination, and helped to draft the manuscript. All authors read and approved the final version of the manuscript.
